# Thyroid Cancer Screening Using Tumor-Associated DN T Cells as Immunogenomic Markers

**DOI:** 10.3389/fonc.2022.891002

**Published:** 2022-05-27

**Authors:** Shahnawaz Imam, Rodis D. Paparodis, Shafiya Imtiaz Rafiqi, Sophia Ali, Azra Niaz, Abed Kanzy, Yara E. Tovar, Mohammed A. Madkhali, Ahmed Elsherif, Feras Khogeer, Zeeshan A. Zahid, Haider Sarwar, Tamanna Karim, Nancy Salim, Juan C. Jaume

**Affiliations:** ^1^ Division of Endocrinology, Diabetes and Metabolism, Department of Medicine, College of Medicine and Life Sciences, University of Toledo, Toledo, OH, United States; ^2^ Center for Diabetes and Endocrine Research (CeDER), University of Toledo, Toledo, OH, United States; ^3^ Private Practitioner, Patras, Greece; ^4^ Windsor University School of Medicine, Cayon St. Kitts West Indies, Saint Kitts and Nevis

**Keywords:** DN T cells, immunogenomic marker, immune editing, PTC, thyroid cancer, active surveillance

## Abstract

**Background:**

Thyroid nodules are an extremely common entity, and surgery is considered the ultimate diagnostic strategy in those with unclear malignant potential. Unfortunately, strategies aiming to predict the risk of malignancy have inadequate specificity. Our group recently found that the microenvironment of thyroid cancer is characterized by an enhanced immune invasion and activated immune response mediated by double-negative T lymphocytes (DN T) (CD3^+^CD4^-^CD8^-^), which are believed to enable or promote tumorigenesis. In the present work, we try to use the DN T cells’ proportion in thyroid fine-needle aspiration (FNA) material as a predictor of the risk of malignancy.

**Methods:**

We recruited 127 patients and obtained ultrasound-guided FNA samples from subjects with cytology-positive or suspicious for malignancy and from those with benign nodular goiter associated with compressive symptoms (such as dysphagia, shortness of breath, or hoarseness), Hashimoto thyroiditis, and Graves’ disease. Out of 127, we investigated 46 FNA samples of patients who underwent total thyroidectomy and for which postoperative histological diagnosis by the academic pathologists was available. We specifically measured the number of cells expressing CD3^+^CD4^-^CD8^-^ (DN T) as a function of total CD3^+^ cells in FNA samples using flow cytometry. We correlated their FNA DN T-cell proportions with the pathological findings.

**Results:**

The DN T cells were significantly more abundant in lymphocytic infiltrates of thyroid cancer cases compared to benign nodule controls (p < 0.0001). When the DN T-cell population exceeded a threshold of 9.14%, of total CD3^+^ cells, the negative likelihood ratio of being cancer-free was 0.034 (96.6% sensitivity, 95% CI, 0.915–1.000, p < 0.0001). DN T cells at <9.14% were not found in any subject with benign disease (specificity 100%). The high specificity of the test is promising, since it abolishes a false-positive diagnosis and in turn unnecessary surgical procedures.

**Conclusion:**

The present study proposes DN T cells’ proportion as a preoperative diagnostic signature for thyroid cancer that with integration of RNA transcriptomics can provide a simplified technology based on the PCR assay for the ease of operation.

## Highlights

1. Double-negative (CD3^+^CD4^-^CD8^-^) T-lymphocyte (DN T) proportion acts as a diagnostic immunogenomic marker of thyroid cancer in FNA samples.
2. DN T-cell proportion greater than 9.14% of CD3^+^ cells is a predictive marker for thyroid cancer with a specificity of 100%.
3. DN T cells present in the tumor immune microenvironment are indicative of progression of disease and could help identify early cancer development.

## Introduction

Thyroid cancer is predicted to become the fourth most common cancer in women by the end of 2030 (1, 2). Approximately 600,000 fine-needle aspirations (FNAs) are performed annually in the United States, and almost a fifth of those (i.e., 120,000 nodules) carry features consistent with atypia of undetermined significance and are suspicious for follicular neoplasms or for thyroid malignancy (3–5). These nodules are commonly subjected to thyroid surgery in order to establish a definitive diagnosis (6–8). These strategies lead to extravagant costs in the care of thyroid cancer that could reach $3.5 billion annually by 2030 (2). Therefore, it becomes imperative to identify preoperative thyroid cancer diagnostic strategies that could enhance our predictive capabilities in order to reduce the number of unnecessary thyroidectomies. In that regard, elegant work in the past 15 years led to breakthrough discoveries that use molecular markers on the FNA cytological material to predict the possibility of malignancy (9). The primary goal of this type of molecular diagnostic tests is to correctly identify benign nodules among those with “undetermined” cytology, thereby decreasing the number of unnecessary diagnostic surgeries. These now conventional predictive markers for thyroid cancer are based on genotypes, and the predictive abilities of these tests are variable and highly dependent on regional differentiated thyroid cancer (DTC) prevalence and genetic signatures (4, 5). In the United States, three main types of molecular tests have been commercially developed over the past decade for cytologically undetermined thyroid nodules: ThyroSeq v3 (University of Pittsburgh Medical Centre and Sonic Healthcare, USA); ThyGeNEXT and ThyraMIR (Interpace Diagnostics, USA); and Afirma Gene Sequencing Classifier (GSC) and Xpression Atlas (Veracyte, USA), with sensitivities of 94%, 93%, and 91% and specificities of 82%, 62%, and 68%, respectively (10). Despite the great sensitivities, the assays lack specificity, causing concern in endocrinologists and patients alike in light of many operations performed for what appeared to be suspicious lesions but proven to be benign after all (11). A high negative predictive value (NPV) is a key feature of any test used to stratify the risk for malignancy and to lead to preoperative correct diagnosis. An approach utilizing cellular profiling of the cancer microenvironment combined with genomics and transcriptomics could enhance the accuracy of the test (12). Recently, our group investigated the role of the immune microenvironment in the pathogenesis of thyroid cancer and found evidence that cancers affect the cellular elements of the immune response of the host, favoring its own survival and propagation, *via* immune editing (13, 14). Characteristic immune cells in the microenvironment of the tumor constitute an individualized “immune signature map” in patients with thyroid cancer (15). In our previous studies, we found that patients operated on for different thyroid surgery indications frequently harbored malignancies when chronic lymphocytic thyroiditis was present in the surgical specimen, and the rate reached 48% when these patients had no preoperative hypothyroidism [euthyroid Hashimoto thyroiditis (EHT)], implying that the specific immune microenvironment in that population could promote tumorigenesis (13, 16). Various other studies have suggested the importance of the immune microenvironment on cancer development that could provide potential biomarkers to improve the reliability and precision of diagnosis and prognosis (17, 18). In the present study, an attempt has been made to establish the frequency of double-negative T lymphocyte (DN T) cells as a marker for thyroid cancer risk and integration of the information derived from immune cellular profiling of FNA of the thyroid cancer microenvironment for the diagnosis/prognosis of thyroid cancer.

## Materials and Methods

### Patient Recruitment

Our Thyroid Multidisciplinary Clinic is a large referral site for thyroid diseases at the University of Toledo Medical Center and its affiliate locations (ProMedica Hospitals). Patients evaluated for thyroid nodules, who were candidates for thyroid surgery, were our recruitment population. Patients referred for thyroid surgery included those with cytology-positive or suspicious for malignancy on fine-needle aspiration (FNA) and those with benign nodular goiter associated with compressive symptoms (such as dysphagia, shortness of breath, or hoarseness), some Hashimoto thyroiditis (HT) (at times painful thyroiditis), and Graves’ disease (GD). A total of 127 patients were recruited for our study based on the above inclusion criteria. The entire patient cohort had ultrasound-guided FNA that was used to extract a tissue sample from an area within and adjacent to a thyroid nodule (tumor microenvironment). Out of the 127 patients, 46 patients had postoperative histological confirmation of the presence of multinodular goiter or HT or GD with or without differentiated thyroid cancer (DTC) or DTC alone [papillary thyroid cancer (PTC) included]. Some of these deidentified cases were recruited at the University of Wisconsin Hospital and Clinics, Madison, WI (kindly provided by Dr. Herb Chen). The results of FNAs as described above were correlated with the postoperative pathology characterized by academic pathologists. For all patients, the following data were collected: gender, age, thyroid autoantibody [thyroid peroxidase antibodies (TPO-Abs)] titers, when available, and the surgical pathology report. Patients on levothyroxine (LT4)-suppressive therapy (when used to prevent the growth of a goiter or thyroid nodules), patients with prior exposure to radioactive iodine, patients with prior thyroid surgery, patients for which post-operative histology was not available or who are yet to have surgery, or patients with incomplete records were all excluded (81 patients). Of note, the mildly hypothyroid patients having no or partial replacement of thyroxine (functional thyroid) were sometimes referred to as “EUHASH” (euthyroid Hashimoto’s) and reported previously for having a high risk for cancer (13, 16).

### Tissue Sampling

**Figure 1:**
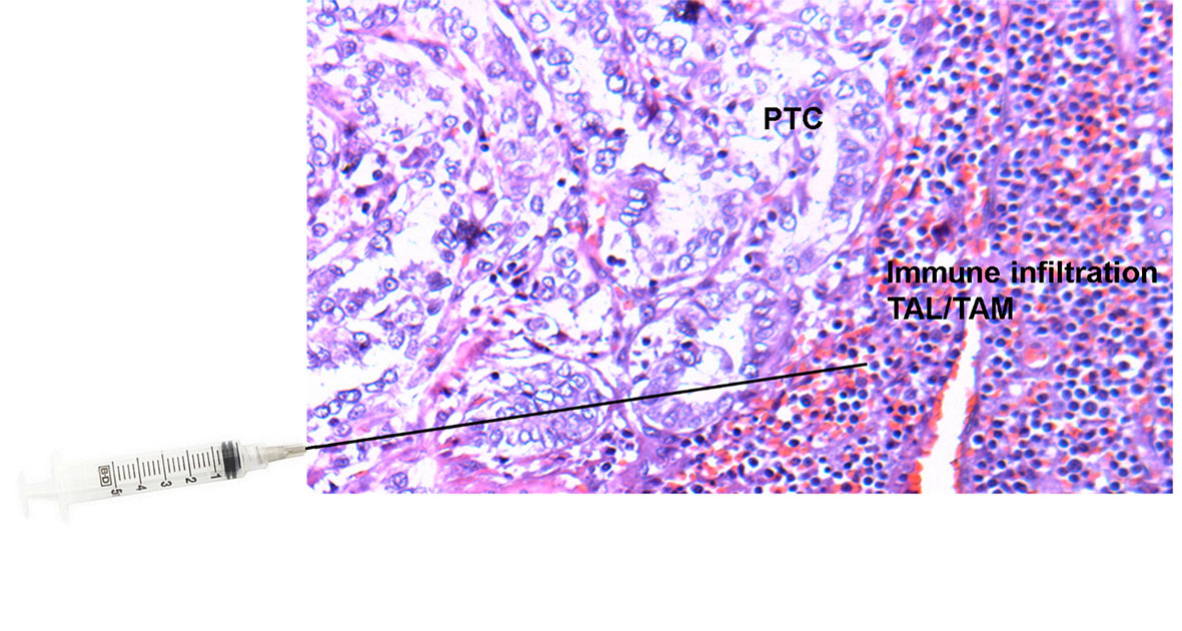
Tissue slide of papillary thyroid cancer (PTC) with adjacent immune cell infiltration [tumor-associated lymphocytes (TALs) and macrophages (TAMs)] stained with Hematoxylin and Eosin (H&E) and superimposed needle illustrating the area within and adjacent to the thyroid nodule from which ultrasound-guided fine-needle aspirations (FNAs) were performed.

Ultrasound-guided FNA was used to extract tissue samples (FNA extra pass) from areas within and adjacent to thyroid nodules (tumor microenvironment). For the evaluation of the nodule microenvironment, needles were directed to the surroundings of the structural abnormality and the FNA sample was subsequently analyzed (**Figure 1**). The extracted material was washed out into appropriate buffers for subsequent flow cytometry profiling of cells and lysate analysis. This procedure is clearly less invasive than a surgical biopsy, leaves no scarring, and does not involve exposure to radiation. The procedure also requires little or no special preparation. The intrathyroidal FNA samples were centrifuged to collect infiltrating leukocytes. The red blood cells (RBCs) in the aspirates were lysed by a brief hypotonic shock. An aliquot of the isolated cells was suspended in RPMI-1640 media containing 10% fetal calf serum (FCS) at 4°C. The cells were stained for surface markers with fluorochrome-conjugated antibodies against human CD3, CD4, CD8, CD16, or isotype controls in serum-containing media, followed by intracellular staining using fluorochrome-conjugated antibodies against human IFNg, IL17, and FOXP3 as described earlier (13, 14). Researchers were blind to the surgical pathology results until statistical analysis.

### Statistical Analysis

The cutoff value for DN T-cell frequency for cancer risk, sensitivity, and specificity of the test was evaluated by the receiver operating characteristic (ROC) curve using MedCalc12.5.0.0 software (Mariakerke, Belgium). Statistical analysis was done using the SAS MIXED procedure (version 9.3, SAS Institute, Inc., Cary, NC, USA). Data were tested for normality by Kolmogorov–Smirnov test and transformed to natural logarithms or ranks as appropriate when not normally distributed. Comparisons between the two groups were done by Student’s t-test or Mann–Whitney U test. Categorical data were analyzed with Fisher’s exact test, and odds ratios (ORs) with 95% confidence intervals (95% CIs) were calculated. The significant difference threshold was set at p ≤ 0.05, and p > 0.05 to p ≤ 0.10 indicated that significance was approached. Data are presented as the mean ± SEM.

## Results

### DN T Cell as a Predominant Population in Thyroid Cancer FNA Samples

**Figure 2:**
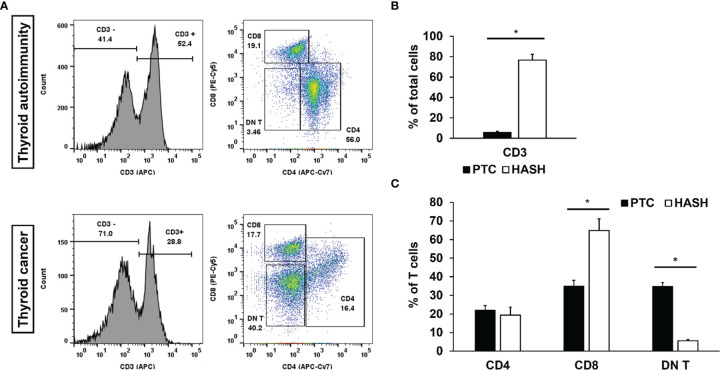
Flow cytometry of Fine Needle Aspiration (FNA) aspirates’ T cells. Histogram of leukocytes and pseudo-color dot plots of lymphocyte specimens from the representative FNA aspirate of the patient with papillary thyroid cancer and hypothyroid Hashimoto thyroiditis **(A)** and bar graphs of statistical analysis of CD3 **(B)** CD4, CD8, and DN T **(C)** [papillary thyroid cancer (PTC), n = 53; Hashimoto (Hash), n = 24]. First, specimens were gated for CD3 and subsequently sorted for CD4, CD8, and DN T. DN T stands for (CD3^+^CD4^-^CD8^-^) T cells. Black bars represent results for FNA specimens from PTC patients, and white bars represent results for FNA specimens from hypothyroid Hash patients. Statistical significance was determined by using t-test: two samples assuming unequal variance (p < 0.05) between the groups. Significance (*) p < 0.05.

Our group previously reported that intrathyroidal lymphocytes accompanying thyroid cancer are tolerant to the presence of the tumor and that these lymphocytes are different from lymphocytes found in autoimmune thyroid disease (14). In the initial screening of 127 thyroid nodules, we used FNA-fluorescence-activated cell sorting (FACS) as a method for diagnosing thyroid cancer. FACS analysis of FNA extra passes revealed that CD3^+^CD4^-^CD8^-^ (DN T) cells were significantly more abundant in lymphocytic infiltrates accompanying thyroid cancer than those present in HT condition (**Figure 2A**). As expected, the proportion of T (CD3^+^) cells present in papillary thyroid cancer (PTC) microenvironments was significantly lower as compared to that of HT (**Figure 2B**). There was no significant difference in the CD4 T-cell count; however, CD8 count dominated in the HT condition (**Figure 2C**). In the same samples, we also confirmed our previous observation that immunoregulatory DN T cells were predominant in PTC. Earlier also, we had reported that in the resected thyroid samples, DN T cells were significantly more abundant in lymphocytic infiltrates of the PTC microenvironment and only traceable in the HT microenvironment (14). That was the first report of immune cell profiling of PTC and of thyroid autoimmunity and first description of a subset of T cells (DN T) present in the setting of thyroid cancer having a “regulatory” role. DN T cells appear to downregulate the proliferation and cytokine production of activated effector T cells present in the tumor microenvironment, contributing to tumor tolerance and active avoidance of tumor immunity (14, 19).

### Comparative Proliferative Cycles of DN T Cells in FNA Samples

**Figure 3:**
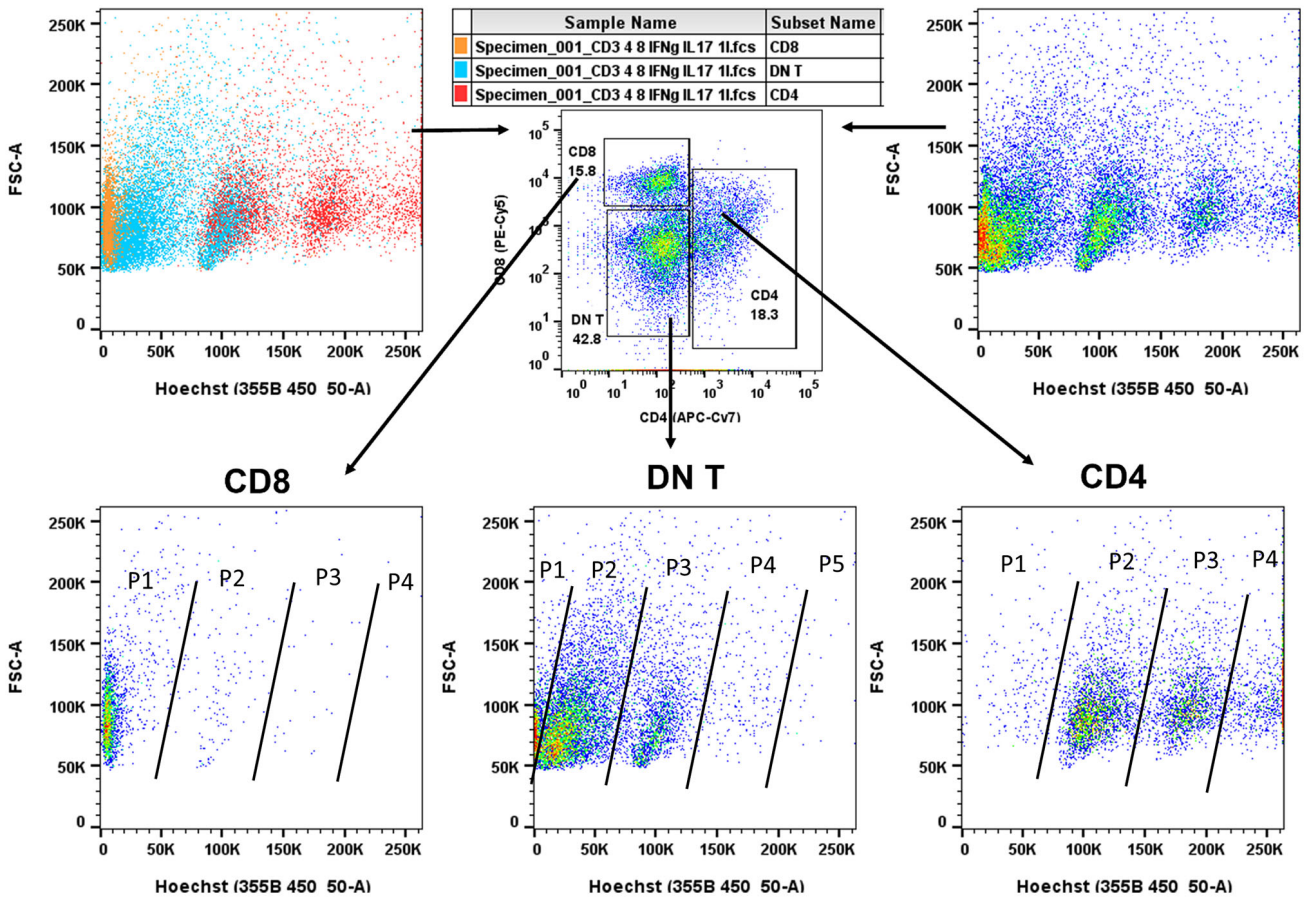
Flow cytometry of Fine Needle Aspiration (FNA) aspirates. Pseudo-color dot plots of lymphocyte specimens from representative FNA aspirates of patients with papillary thyroid cancer (PTC). First, specimens were gated for CD3 and subsequently sorted for CD4, CD8, and DN T cells. Lymphocytes were also stained with Hoechst 33342 (DNA staining to follow DNA replication cycle, top panels, left, cell type colored, right, cell proliferation colored). As shown (bottom panels), DN T (middle)-cell population was predominant and in high DNA replicative state as compared to cytotoxic CD8 T (left)-cell population (P1–P5, Population cycle). Although CD4 T (right) cells were also in high DNA replicative state, the proportion of proliferating cells was lower at each replicative cycle (P1–P4) compared to DN T populations (P1–P5). Of note, CD4 T-cell populations (P1–P4) include proliferating Treg cell population known to be present in the tumor microenvironment as previously reported (14). Although we observed a considerable proportion of cytotoxic CD8 T cells, their proliferative capacity was limited may be because of a high DN T regulatory-cell presence.

In the present study, we systematically characterized the lymphocytic microenvironment of human FNA thyroid specimens. The DN T-cell population predominated and was in high proliferative cycle as compared to cytotoxic CD8^+^ T-cell population in the cancer microenvironment. Although CD4 T cells seem proliferative too, their proportion of total CD3^+^ T cells was lower as compared to DN T. The high proliferative CD4 T-cell state, albeit in low absolute numbers, may represent proliferating regulatory T (Treg) cell population (25% of CD4 T cells) known to be present in the microenvironment of cancer, as we have reported in our previous paper (14) **(** **Figure 3**). We observed a considerable cytotoxic CD8 population presence, but its proliferative cycle was limited may be because of high DN T cell presence and its regulatory capacity (**Figure 3** lower first panel) (14).

### Confirmatory Diagnostic Value of Fine Needle Aspiration (FNA)-Fluorescence-Activated Cell Sorting (FACS) and Statistical Validation of DN T–Cancer Association

**Figure 4:**
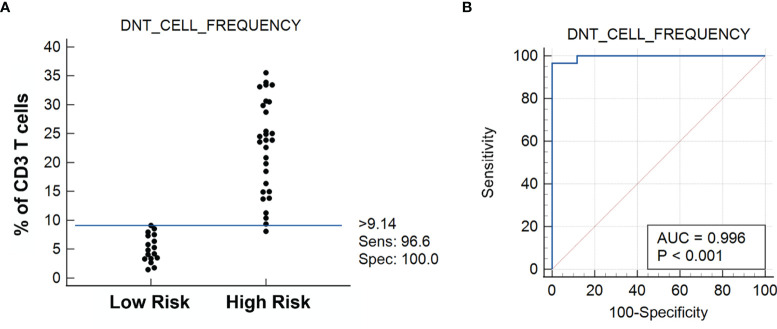
**(A)** Dot diagram shows the DN T-cell frequency vs. the risk for cancer as established by the receiver operating characteristic (ROC) curve analysis showing the sensitivity and specificity of the test (n = 46) **(B)**. Statistically, >9.14% DN T cells of total T cells determine the risk for cancer, with all cases under Low Risk being benign lesions and all cases under High Risk being cancerous lesions. Please note that 1 case under High Risk (the only medullary thyroid carcinoma case in the cohort study) falls below the 9.14% cutoff limit.

We systematically characterized the lymphocytic microenvironment of FNA samples and compared them with the postsurgical pathological diagnosis of the same resected thyroid specimens. In this study comprising 46 FNA samples, the DN T-cell frequency in FNA was used to predict the risk for thyroid cancer, with the prediction being validated by postoperative pathological diagnosis (**Supplementary Table S1**). The hypothesis was tested using MedCalc 12.5.0.0 software (Mariakerke, Belgium). The cutoff value for DN T-cell frequency was evaluated by the ROC curve. Statistically, a patient is at higher risk for cancer if the frequency of DN T cells is >9.14%, the sensitivity of the test being 96.6% and specificity 100% (**Figure 4**). ROC analysis of data revealed an area under the curve (AUC) of 0.996 (95% CI, 0.915–1.000). The high specificity of the test is promising, since it abolishes the false-positive diagnosis and prevents unnecessary surgical procedures. Thus, if the quantity of DN T cells exceeds the defined threshold of 9.14%, it indicates a prospective cancer presence. The negative likelihood ratio (-LR) of the test is 0.034, a relatively low likelihood ratio that significantly decreases the probability of a false-positive diagnosis. The current molecular testings based on the identification of mutations reduce the risk of cancer rather than guaranteeing the absence of cancer (1). Therefore, our test ensures a 100% specificity that prevents a benign thyroid nodule from undergoing surgery.

We also correlated the postoperative pathological diagnosis with the FNA DN T-cell frequency and the clinical history. As previously reported, the risk of developing thyroid cancer was higher in patients with a silent form of autoimmune thyroid disease, EHT (or EUHASH), as compared to GD and with non-autoimmune thyroid diseases (non-AITDs). The predicted risk was especially pronounced in patients with functional thyroids and undetectable/low titers of TPOs as described for EHT while diminished in patients with full thyroid failure and high TPO antibody titers [**Supplementary Table S1** and (13)].

### Presence of Differential Natural Killer (NK) Cell Activity May Decide the Fate of Thyroid Cancer

**Figure 5:**
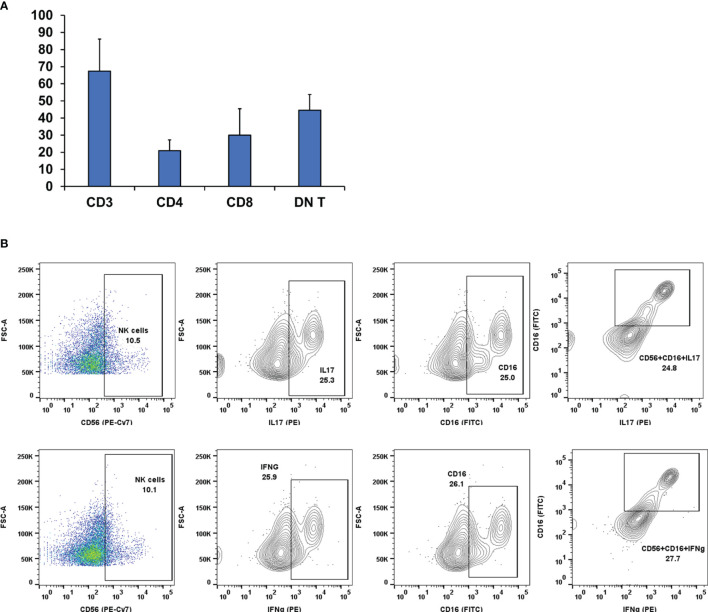
**(A)** Lymphocyte distribution in Fine Needle Aspiration (FNA) samples. In some samples (n = 4), there was a high CD3 count along with a high DN T-cell count but were non-cancerous. Therefore, we probed the samples for other leukocyte types [i.e., Natural Killer (NK)]. **(B)** Pseudo-color dot plots of lymphocyte specimens from representative FNA aspirates of patients with background Hashimoto thyroiditis. First specimens were gated for CD3^-^ and subsequently sorted on the basis of surface staining CD56, CD16, and intracellular staining Interleukin 17 (IL17) (**B**, top panels) and Interferon gamma (IFNg) (**B**, bottom panels). The flow cytometry analysis revealed that in these samples, NK (CD3^-^CD56^+^) cells have a high expression of CD16^+^IFNg and CD16^+^IL17. Activated NK cells expressing CD16/IFNg/IL17 have a strong immune activator function that might drive CD3 cell proliferation.

In some FNA samples (4 out of 127), we observed that the percentage of CD3 cells out of the total cell count was very high as in HT despite having a high DN T-cell population unlike the other FNA samples of HT (**Figure 5A**). Knowing that natural killer (NK) cells may modulate the Treg-cell balance, we investigated the functional characteristics of the NK cell population as described in our previous paper (13). We analyzed the activation status of NK cells with different cell surface (CD16) and intracellular markers (IFNg, IL17) using fluorescent conjugated monoclonal antibodies for flow cytometry. The flow cytometry analysis revealed that in these 4 samples, NK (CD3^-^CD56^+^) cells had a high expression of CD16^+^IFNg^+^ and CD16^+^IL17^+^ (**Figure 5B**). Activated NK cells expressing CD16/IFNg/IL17 have a strong immune activator function (20, 21) that may induce plasticity changes in tumor-associated macrophages [TAMs/type 2 macrophages (M2)] toward the type 1 macrophage (M1) phenotype that may convert the tumor microenvironment toward a pro-inflammatory state and allow for active avoidance of thyroid cancer as described previously (13).

## Discussion

Thyroid cancer is one of the most common malignancies worldwide and could become the fourth most common cancer overall by 2030. Its rising incidence is thought to relate to the rapidly expanding use of point-of-care thyroid ultrasound (22), even though this explains only part of that rise (23). Thousands of thyroid nodules are subjected to surgery based on a thyroid FNA procedure and subsequent cytology. In addition to clearly malignant cytological appearance of FNAs, thyroid surgery is frequently used as a diagnostic strategy in nodules with atypia of undetermined significance/follicular lesion of undetermined significance, those suspicious for follicular neoplasm, and a certain number with non-diagnostic FNAs. Thyroid surgery in these cases is associated with a risk for malignancy ranging from 15% to 30%, rendering the majority of the surgical procedures unnecessary. Given the rapidly rising costs in health care overall, and those related to thyroid cancer care (1), a preoperative diagnostic maneuver, with optimal positive predictive value (PPV) and negative predictive value (NPV), could be invaluable in allowing for the needed appropriate care and diminishing the costs associated with this condition.

Thyroid cancer prediction with the use of molecular profiling techniques has been attempted in the past decade with some success. The third generation of the ThyroSeq classifier (University of Pittsburgh Medical Centre and Sonic Healthcare, USA) uses DNA and RNA-based next-generation sequencing analyzing 112 genes for a variety of genetic alterations using a genomic classifier (GC) to separate malignant lesions from benign lesions. In a landmark study (24) of 286 patients with indeterminate nodules, a specificity of 82% and a PPV of 66% were reported, thus allowing for a reclassification of the malignant potential and substantial reduction in unnecessary thyroid surgeries. In 2012, the Afirma Gene Sequencing Classifier was introduced (25), which assessed the expression of 167 genes in the FNA specimen, yielding a sensitivity of 92% but a specificity of only 52%. More recently and from the same company, the Xpression Atlas (Veracyte, USA) (26) uses mRNA expression analysis and achieves a sensitivity of 92% and a specificity of 58%. In 2016, a hybrid profiling strategy, ThyraMIR, was introduced by Wylie et al. (27) using 17 validated oncogenic gene alterations in the BRAF, RAS, RET, or PAX8 genes and the expression of multiple microRNAs deemed oncogenic. Reportedly, it produced a 75% cancer detection rate and 86% accurate assessment of benign lesions. Recently, a novel predictive strategy emerged, ThyGeNEXT (28), which also uses a hybrid technique, incorporating data from an expanded mutation panel in combination with microRNA classification. This classifier divides the results into low, medium, and high risk, yielding a 53% PPV in patients at the high-risk group.

These diagnostic tools produce molecular signatures unique to each nodule and allow for better characterization of the malignant risk but do not achieve optimal specificity. In addition, the cost related to these techniques is remarkably high, even though cost-effectiveness analyses revealed some financial benefits when they are used instead of surgery. Our group has been studying for several years the role of the immune system in the risk for differentiated thyroid cancer in terms of clinical parameters, biochemical markers, and the particular effects of chronic lymphocytic thyroiditis. Out of many features of the immune response that we studied (13), the DN T (CD3^+^CD4^-^CD8^-^) cells stood out as a potential mediator of tumor cell proliferation and immune response tolerance (14). Their numbers were 20× higher in the presence of thyroid cancer as compared to those found in chronic lymphocytic thyroiditis or benign multinodular goiter, and stimulation with phorbol 12-myristate 13-acetate/ionomycin left them unexpectedly unchanged (14). Given the magnitude of the difference in the numbers of these cells, we designed the present study to assess whether the proportion of these cells in a thyroid FNA could be useful as a diagnostic marker for thyroid cancer.

An immune score based on immunological parameters of the tumor microenvironment can be of diagnostic and prognostic value. The type, density, and location of immune cells within the tumor microenvironment definitively play a central role in disease progression (29). In general, the tumor-associated immune cells can be divided into two types: tumor-antagonizing and tumor-promoting immune cells. Tumor-antagonizing immune cells consist of effector T cells, NK cells, dendritic cells (DCs), and M1 polarized macrophages; while tumor-promoting immune cells include lymphoid lineage Treg cells, myeloid origin TAMs (M2 TAMs), tumor-associated neutrophils (TANs), tolerogenic DCs (tDCs), and other kinds of suppressive cells. The major suppressors that help in creating a tumor-promoting microenvironment are all kinds of Treg cells (30).

The present study focuses on establishing a phenotypic marker based on immune cells in the tumor microenvironment using FNA. It comprises a method to extract a tissue sample from an area within and adjacent to thyroid nodules and a relative quantification of DN T cells determining whether the thyroid nodules are likely to be cancerous. DN Ts are a subset of CD3^+^ cells having either αβ or γδ T-cell receptors (TCRs), devoid of mature T-cell markers (CD4, CD8, and CD56). These cells constitute approximately 1%–5% of circulating T cells (31) exhibiting duality of function being tolerogenic in transplants (preventing graft rejections) and sometimes cytotoxic against leukemia and some other blood neoplasms (32–34).

Since we are proposing DN T-cell expansion as a criterion for solid tumor malignancy, awareness that they may be markedly expanded in some rare blood cancers (35) is important to avoid misdiagnosis if solid and blood cancers were to coexist. DN T dominance was also found in cytofluorimetric analyses of tissues from many tumor types such as melanomas, renal cell carcinoma, glioblastoma (36), and melanoma-invaded lymph nodes compared to negative lymph nodes (37). De Tullio et al. (38) reported that in Hodgkin’s lymphoma, αβ-DN Ts were significantly increased as compared with other histotypes, suggesting an important role in the development and progression of Hodgkin’s lymphoma. Another group of researchers demonstrated that *ex vivo* expanded DN Ts exert an antitumor activity, hinting at a possible way for adoptive cell therapy (38, 39).

In our present study, DN T cells were quantified from FNA of thyroid specimens and found in higher proportions in PTC samples. The DN T-cell count was up to 40% of the total T cells, showed an atypical proliferation pattern, and possibly had an immunomodulatory role and consecutively downregulated the CD4 and CD8 T-cell population as shown previously (14). CD4 T-cell population was found in similar proportion in thyroid cancer as in HT, but the subtype distribution does not seem to be the same. We presumed that the CD4 population depicted in **Figure 3**, lower right panel, might be mostly Treg cells. In our previous paper, we described that 25% of CD4 T cells are CD25^+^FoxP3^+^ (14) in the tumor microenvironment. We speculate that these regulatory cells (Tregs and DN T cells) synergistically regulate the effector (CD4/CD8) T cells.

Our group already identified DN T cells as a dominant cell type in the thyroid cancer microenvironment, associated with downregulating the proliferation of activated effector T cells (14). Later on, a commentary published by Weber (19) recommended reanalyzing the findings to identify the proportion of DN T cells in patients with aggressive PTC and with indolent stages. Taking a cue from that, the present study focused on diagnosing thyroid cancer based on DN T cells, defining a DN T-cell proportion of >9.14% as indicative of thyroid cancer. The prediction of thyroid cancer based on preoperative FNA DN T-cell population was verified with postoperative histological confirmation of the presence of thyroid cancer. Furthermore, the observed results were validated statistically by ROC curve analysis using MedCalc software. The ROC curve is a fundamental tool for diagnostic test evaluation of the accuracy of a test to discriminate diseased cases from normal cases (40, 41). The ROC curve analysis revealed a 96.6% sensitivity and 100% specificity for a DN T-cell frequency >9.14% as indicative of a high risk of thyroid cancer. The postoperative confirmatory histologic diagnosis was held as a reference standard for the test. A likelihood ratio expresses the relative odds that a given level of a diagnostic test result would be expected in a patient with (as opposed to one without) the target disorder (42). Our test had a negative likelihood value of 0.034. Of note, one cancer sample had a DN T population of <9.41% but was diagnosed as medullary thyroid cancer (MTC) with a DN T-cell population of 8.12% T cells.

The choice of optimal sensitivity and specificity for a test is the most challenging aspect in the evaluation of a diagnostic test and determined by the end use of the test. The present criterion of 9.14% cutoff at 100% specificity highlights the test ability toward predicting benign nodules and identifies the test as a “rule-out test.” Even with current molecular testing, a significant number of intermediate thyroid cytology patients undergo diagnostic surgery, but only 30% of those patients are diagnosed with malignant nodules (1). Unnecessary ~70% thyroidectomy rate for benign lesions is not ideal; hence, a rule-out test will directly influence cost-effectiveness and patient satisfaction.

Notably, in the United States, higher-income groups have a higher incidence of thyroid cancer (43) possibly as the result of overdiagnosis (44). A study unveiled a financial disadvantage of privately insured patients and raised a concern of increased probability of total thyroidectomy, lymphadenectomy, and postoperative radioactive iodine (RAI) therapy in privately insured patients (45). Moreover, in the United Kingdom, the initial treatment including diagnostics, surgery, and adjuvant RAI accounts for £473 million (or 41% of the total annual cost), and £428 million (37% of total costs) is taken by the management of the follow-up of thyroidectomized patients (1).

We also observed that in some samples (**Figure 5A**), there was a high DN T-cell count and a high CD3 count. Usually, the CD3 count is on the lower side in case of PTC (**Figure 2B**). Therefore, we investigated the immune cell profiling of this patient subcohort (n = 4) and found that not only DN T-cell population but also the presence and activity of NK cells and NK cells expressing CD56^+^CD16^+^IFNg^+^IL17 had a cascade effect on the plasticity of macrophages (13). M1s are the pro-inflammatory macrophages having an anti-cancerous role in the tumor microenvironment, whereas M2s have a role in creating a protective immune barrier for solid tumors (13). The role of NK cell-mediated macrophage plasticity in case of thyroid cancer has been well documented in our previous paper (13). Activated NK cells can drive M1 differentiation, which in turn is cytotoxic to the cancer cells and downregulates M2s (13). The production of IFNg may act as a major inductor of NK cell cytotoxicity toward tumor cells. Cancer seems capable of disrupting the M1 dominance by tipping the macrophage plasticity balance toward the M2 phenotype; hence, the progression/occurrence of thyroid cancer is macrophage plasticity dependent, which in turn is dependent upon the microenvironment/NK dominance.

**Figure 6:**
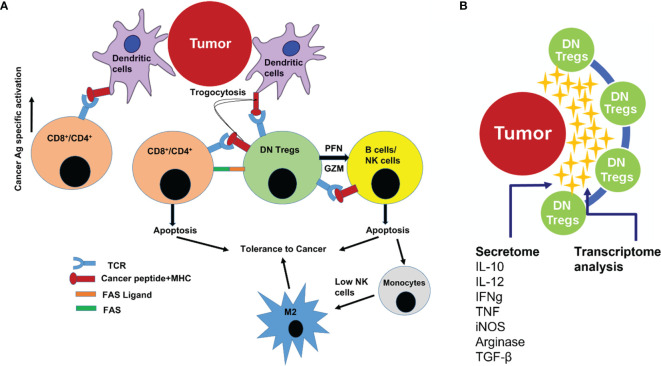
**(A)** Cartoon illustration showing how DN T cells regulate the proliferation and effector T-cell function in the tumor microenvironment. DN T cells cause peripheral deletion of activated T cells through repeated T Cell Receptor (TCR) stimulation (by exhaustion). DN T cells also induce apoptosis of B cells and Natural Killer (NK) cells (by granzyme and perforin cytotoxicity). Absence of, or a low count of, active NK cells leads to macrophage plasticity activation that allows macrophage subtype M0 to differentiate to M2 phenotype (precancerous/cancerous-associated phenotype). **(B)** Cartoon illustration showing that the interplay between DN T cells in the tumor microenvironment was mediated also through several secretory cytokines and chemokines (secretome), allowing for the use of transcriptome analysis to pinpoint the onset/progression of thyroid cancer.

Finally, DN T cells acquire regulatory potency through prior activation by exposure to cognate antigen from DCs through a mechanism known as trogocytosis, which implies that DN T cells activate themselves with the same Major Histocompatibility Complex (MHC)-restricted peptide as CD4/CD8 T cells (34). That is how DN T cells prime themselves to a specific antigen in order to exert antigen-specific regulatory phenomena. It also has been reported in humans that Tregs have direct inhibitory effects on the antigen-presenting function of monocytes/macrophages, as shown by their reduced capacity to stimulate autoantigen- or alloantigen-specific T-cell responses (46, 47). DN Tregs can cause downregulation of the costimulatory molecules CD80 and CD86 on DCs (48). That is how DN T cells downregulate the antigen-specific proliferation of effector cells (CD4/CD8) T cells (14). Such immunoediting capacity in the tumor microenvironment may lead to better survival of the developing PTC (**Figure 6A**).

DN T cells also express perforin and granzyme that kill NK cells (49) in the tumor microenvironment. Depleted NK cell population may favor the differentiation of monocytes (M0) toward M2 phenotype macrophages. Therefore, DN T cells may have the potential to block the NK-generated pro-inflammatory immune environment and favor cancer cell survival (13). DN T cells may also suppress NKs *via* membrane-bound transforming growth factor beta (TGF-β) (50). Thus, NK cell function inversely correlates with DN T-cell frequency in thyroid cancer patients (13). Human Treg cells express membrane-bound TGF-β, which may also directly inhibit NK cell effector function and downregulate NKG2D receptors on the NK cell surface (50). These findings support a role for DN T cells as Treg cells in blunting the NK cell arm of the innate immune system. Furthermore, DN T cells, by restricting the availability of IL-2 in the cancer microenvironment, may prevent the NKs from proliferating, secreting IFN-g, and enhancing missed self-recognition (51). Not only that, DN T cells are able to kill both allogeneic and syngeneic B cells *via* a perforin-dependent pathway (52). These findings indicate that DN T cells can suppress immune responses by exerting an effect on APCs as well (**Figure 6A**).

### Conclusion

In conclusion, our study proposes a cutoff of >9.14% based on quantification of DN T cells as an immunogenomic marker for early diagnosis of thyroid cancer with 100% specificity. We propose that instead of a linear evaluation, integration of clinical evidence, ultrasound assessment, cytological parameters, and DN T-cell frequency can assist in arriving at a conclusive diagnosis in case of an indeterminate thyroid cytology. This analysis provides not just a diagnostic marker but also a predictive tool for severity/improvement of cancer in thyroid cancer active surveillance patients.

### Future Directions

The present finding is aimed to identify a diagnostic cell profile to reduce the ~70% unnecessary thyroid surgeries where FNA cytology results are inconclusive. The cellular interplay within the tumor immune microenvironment is mediated through several secretory cytokines and chemokines (secretome), which influence the onset/progression of thyroid cancer. Thus, it becomes important to understand the functional interactions between the tumor microenvironment and thyroid cancer at the humoral level as well. High-throughput comprehensive gene expression analysis of patients’ samples using FNA aspirates (especially from the tumor microenvironment) will help to annotate and track dynamic changes in gene expression at different cellular levels in all patients having DN T-cell populations >9.14%. This multitranscript (RNA/comprehensive gene expression/immune cells/humoral mediators) approach will unveil which genes and pathways correlated with the immunological cell expression patterns before, during, and/or after the onset of PTC (**Figure 6B**). Modeling tools will be generated to investigate parameters predictive of PTC. The in-depth multitranscriptome data analysis will finally enable us to adopt the immunogenomic markers on a PCR-based platform.

## Data Availability Statement

The original contributions presented in the study are included in the article/**Supplementary Material**. Further inquiries can be directed to the corresponding authors.

## Ethics Statement

The studies involving human participants were reviewed and approved by the Institutional Review Board (IRB), University of Toledo. The patients/participants provided their written informed consent to participate in this study.

## Author Contributions

SI: Conception, designing, and executing the experiments. Analysis, interpretation of the data. Drafting and approval of the final version of the article. JCJ: Conception, designing, analysis, and interpretation of the data,, critical review, and approval of the final version of the article. RP: Conception and designing of the study. SR: Statistical analysis and drafting and reviewing the article. SA: Patient recruitment and procurement of FNA samples. AN: Patient recruitment, procurement of FNA samples, and compilation of pathological findings. AK: Patient recruitment and procurement of FNA samples. YT: Patient recruitment and procurement of FNA samples. MM: Patient recruitment and procurement of FNA samples AE: Patient recruitment and procurement of FNA samples. FK: Patient recruitment and procurement of FNA samples. ZZ: Analysis of compiled pathological findings. HS: Assisting in *in vitro* experimentation. TK: Assisting in *in vitro* experimentation. NS: Data collection and analysis and drafting of the article. All authors contributed to the article and approved the submitted version.

## Funding

The work was funded by the University of Toledo, College of Medicine and Life Sciences.

## Conflict of Interest

The authors declare that the research was conducted in the absence of any commercial or financial relationships that could be construed as a potential conflict of interest.

## Publisher’s Note

All claims expressed in this article are solely those of the authors and do not necessarily represent those of their affiliated organizations, or those of the publisher, the editors and the reviewers. Any product that may be evaluated in this article, or claim that may be made by its manufacturer, is not guaranteed or endorsed by the publisher.
