# Supplementary material for: Thyroid Cancer Screening Using Tumor-Associated DN T Cells as Immunogenomic Markers
Source: Front Oncol. 2022 May 27;12:891002. doi: 10.3389/fonc.2022.891002 (PMC9186057; doi:10.3389/fonc.2022.891002)
Supplement: Supplementary file 1 [file Table_1.docx]

| **Study ID** | **Pre-operative diagnosis** | **Thyroid Function Status** | **Levothyroxine supplementation dose** | **DNT Freq.** | **Risk for cancer based on DNT** | **Pathological Diagnosis post -operative** |
| --- | --- | --- | --- | --- | --- | --- |
| 1 | FLUS | Mildly hypothyroid TPO - | 0.60 mcg/Kg | **30.64** | High risk | PTC |
| 2 | FLUS | Mildly hypothyroid TPO - | 0.22 mcg/Kg | **24.89** | High risk | PTC |
| 3 | FLUS | Mildly hypothyroid TPO - | 0.46 mcg/Kg | **25.43** | High risk | PTC |
| 4 | FLUS | Mildly hypothyroid TPO - | 0.73 mcg/Kg | **33.42** | High risk | PTC |
| 5 | FLUS | Mildly hypothyroid TPO - | 0.63 mcg/Kg | **23.87** | Very high risk | HASH-PTC |
| 6 | PTC | Mildly hypothyroid - TPO + | 1.17 mcg/Kg | **20.8** | High risk | PTC - HASH |
| 7 | PTC | Mildly hypothyroid TPO - | 0.82 mcg/Kg | **29.9** | Very high risk | PTC - HASH |
| 8 | PTC | Mildly hypothyroid TPO + | 0.77 mcg/Kg | **35.57** | High risk | PTC - HASH |
| 9 | FLUS | Mildly hypothyroid TPO - | 1.02 mcg/Kg | **23.57** | High risk | PTC - HASH |
| 10 | FLUS | Mildly hypothyroid TPO + | 0.68 mcg/Kg | **16.34** | High risk | PTC - HASH |
| 11 | FLUS | Mildly hypothyroid TPO + | 0.88 mcg/Kg | **14.97** | High risk | PTC - HASH |
| 12 | Goiter | Euthyroid - TPO + | None | **10.41** | High risk | EUTHYROID HASH |
| 13 | Dysphagia | Mildly hypothyroid TPO + | 1.22 mcg/Kg | **4.09** | Low risk | HASH |
| 14 | Dyspnea, OSA | Mildly hypothyroid TPO + | 1.06 mcg/Kg | **5.3** | Low risk | HASH |
| 15 | Dysphagia, SOB | Fully hypothyroid TPO + | 2.87 mcg/Kg | **3.342** | Low risk | HASH |
| 16 | FLUS | Euthyroid TPO - | None | **23.87** | Very high risk | PTC - HASH |
| 17 | PTC | Fully hypothyroid - TPO + | 1.7 mcg/Kg | **19.8** | High risk | PTC - HASH |
| 18 | PTC | Euthyroid - TPO - | None | **28.7** | Very high risk | PTC - HASH |
| 19 | PTC | Mildly hypothyroid TPO + | 0.67 mcg/Kg | **33.47** | High risk | PTC - HASH |
| 20 | Compressive symptoms | Mildly hypothyroid TPO - | 1.07 mcg/Kg | **13.7** | High risk | PTC - HASH |
| 21 | FLUS | Mildly hypothyroid TPO + | 0.68 mcg/Kg | **6.34** | Low risk | HASH |
| 22 | FLUS | Mildly hypothyroid TPO + | 0.88 mcg/Kg | **4.87** | Low risk | HASH |

**Table 1**

| 23 | MNG | TPO -ve | None | **2.65** | Low risk | MNG |
| --- | --- | --- | --- | --- | --- | --- |
| 24 | Goiter | Euthyroid HASH - high TPO titer | None | **8.51** | Low risk | HASH - Benign |
| 25 | Dysphagia | Mildly hypothyroid high TPO titer | 1.22 mcg/Kg | **7.30** | Low risk | HASH - Benign |
| 26 | Graves | TPO +ve | None | **3.50** | Low risk | Graves |
| 27 | Dyspnea, OSA | Mildly hypothyroid high TPO titer | 1.06 mcg/Kg | **22.60** | High risk | PTC |
| 28 | Graves | TPO +ve 1:100 | None | **4.23** | Low risk | Graves |
| 29 | Follicular Neoplasm | TPO +ve | None | **14.90** | High risk | PTC |
| 30 | Dysphagia, SOB | Fully hypothyroid - High TPO titer | 2.87 mcg/Kg | **7.50** | Zero risk | HASH - Benign |
| 31 | PTC | TPO -ve | None | **33.88** | High risk | PTC |
| 32 | MTC | Not available | None | **8.12** | **Low risk** | MTC |
| 33 | MTC | TPO +ve 1:100 | None | **18.5** | High risk | HCC |
| 34 | FLUS | Euthyroid HASH - TPO negative | None | **9.387** | High risk | PTC - HASH |
| 35 | PTC | Fully hypothyroid - High TPO titer | 1.7 mcg/Kg - | **13.80** | High risk | PTC - HASH |
| 36 | Dysphagia | TPO -ve | None | **7.93** | Low risk | MNG |
| 37 | PTC | Euthyroid HASH - low TPO titer | None | **24.54** | High risk | PTC - HASH |
| 38 | PTC | Mildly hypothyroid - High TPO titer | 0.67 mcg/Kg - High risk | **25.00** | High risk | PTC - HASH |
| 39 | PTC | Mildly hypothyroid Low TPO titer | 0.3 mcg/Kg | **11.27** | High risk | PTC |
| 40 | Compressive symptoms | Mildly hypothyroid - Low TPO titer | 1.07 mcg/Kg | **30.47** | High risk | PTC |
| 41 | Cellular nodule | TPO -ve | None | **3.355** | Low risk | MNG |
| 42 | FLUS | Hypothyroid - High TPO titer | Unknown | **5.788** | Low risk | HASH |
| 43 | Benign | Not available | None | **1.75** | Low risk | Benign |
| 44 | FLUS | Mildly hypothyroid – TPO -ve | 0.88 mcg/Kg | **9.14** | High risk | HASH |
| 45 | Goiter | TPO -ve | None | **1.46** | Low risk | MNG |
| 46 | PTC | TPO -ve | None | **33.12** | High risk | PTC |
